# Assessing antimicrobial resistance profiles of Salmonella enterica in the pork production system

**DOI:** 10.1099/jmm.0.001894

**Published:** 2024-09-25

**Authors:** Teerarat Prasertsee, Sakaoporn Prachantasena, Phakawat Tantitaveewattana, Podjanakorn Chuaythammakit, Ben Pascoe, Prapas Patchanee

**Affiliations:** 1 Faculty of Veterinary Science, Prince of Songkla University, Hat-Yai, Songkhla, Thailand; 2 Department of Biology, Ineos Oxford Institute for Antimicrobial Research, University of Oxford, Oxford, UK; 3 Faculty of Veterinary Medicine, Chiang Mai University, Muang, Chiang Mai, Thailand; 4 Veterinary Academic Office, Faculty of Veterinary Medicine, Chiang Mai University, Muang, Chiang Mai, Thailand

**Keywords:** antimicrobial resistance (AMR), multidrug resistance (MDR), pig production, *Salmonella enterica*, prevalence

## Abstract

**Introduction.***Salmonella enterica* is a significant enteric pathogen affecting human and livestock health. Pork production is a common source of *Salmonella* contamination, with emerging multidrug resistance (MDR) posing a global health threat.

**Gap statement.***Salmonella* contamination and antimicrobial resistance (AMR) profiles in the pig production chain are underreported.

**Aim.** To investigate the prevalence of *S. enterica* in the pig production chain and characterise their AMR profiles.

**Methodology.** We collected 485 samples from pig farms, a standard pig abattoir and retail markets in Patthalung and Songkhla provinces in southern Thailand. Antimicrobial susceptibility testing was performed on these samples, and AMR profiles were determined.

**Results.***S. enterica* was detected in 68.67% of farm samples, 45.95% of abattoir samples and 50.67% of retail market samples. Analysis of 264 isolates, representing 18 serotypes, identified *S. enterica* serotype Rissen as the most prevalent. The predominant resistance phenotypes included ampicillin (AMP, 91.29%), tetracycline (TET, 88.26%) and streptomycin (STR, 84.47%). Over 80% of isolates showed resistance to three or more antimicrobial classes, indicating MDR. The AMP–STR–TET resistance pattern was found in nearly 70% of all MDR isolates across the production chain.

**Conclusions.** The high prevalence of MDR is consistent with extensive antimicrobial use in the livestock sector. The presence of extensively resistant *S. enterica* highlights the urgent need for antimicrobial stewardship. Strengthening preventive strategies and control measures is crucial to mitigate the risk of MDR *Salmonella* spreading from farm to fork.

**Impact Statement**

This study identified the widespread presence of *Salmonella enterica* and the dissemination of antimicrobial resistance throughout the pork production chain in Southern Thailand. The high prevalence of multidrug-resistant *Salmonella* is a public health risk, and our evidence supports the need for increased monitoring and control measures to protect public health.

## Introduction

The World Health Organization (WHO) estimates that almost 1 in 10 people fall ill from foodborne diseases each year, and 33 million healthy life years are lost [1,2]. Foodborne illness can be severe, and diarrhoeal disease affects up to 550 million people each year, including 220 million children under the age of 5 years. Non-typhoidal *Salmonella* (NTS) typically induces acute gastroenteritis, primarily manifesting as diarrhoea in warm-blooded animals [3,4]. More than 2600 serological variants of *Salmonella enterica* have been identified [5], with over 153 million NTS infection cases identified worldwide in 2010 alone and over 56 000 associated deaths [6]. Regional public health surveillance estimates of NTS infections vary globally [7], with more than 1.35 million cases reported annually in the USA between 2004 and 2018 [8], 22.8 million cases in Southeast Asia in 2010 [9], 90 000 cases in the European Union in 2014 [10] and 185 cases per 100 000 population recorded in Australia between 2001 and 2016 [11].

It is important to consider *Salmonella* transmission networks from a One Health perspective, as most human infections can be attributed to spread within the food supply chain [12,13]. NTS infections have been linked with contaminated food products, such as pork, poultry meat and egg-based products [14,16]. Pig farming and the pork production process serve as a significant reservoir for *Salmonella* transmission to humans, and several *Salmonella* serovars have been shown to infect pigs and survive through the pork production chain [17]. *Salmonella* contamination is especially high in Southeast Asian supply chains, with detection levels as high as 80% on pig farms and markets in the Mekong Delta region [18,20]. In addition, detection levels increased from 29 to 66% in European pig production between 2008 and 2015 [21] contributing to significant global public health concerns.

Zoonotic transmission of *Salmonella* spp. between livestock carriage and humans causes Salmonellosis [22], and gastroenteritis is usually self-limiting; however, vulnerable populations, such as children under 5 years, the elderly and immunocompromised individuals, may experience severe complications, which require antibiotic treatment [23]. Fluoroquinolones and extended-spectrum cephalosporins are the first-line antimicrobial agents for the treatment of severe human salmonellosis [24], but these antibiotics are also used in livestock farming [25]. Antimicrobial resistance (AMR) is increasing in clinical treatment options, including ampicillin (AMP), ciprofloxacin (CIP), chloramphenicol (CHL), florfenicol, sulfamethoxazole–trimethoprim and tetracycline (TET) [26]. In Thailand, using antibiotics as a growth promotor has been restricted for use in food-producing animals since 2015 by the Ministry of Agriculture [27]. However, antibiotics are frequently used *en masse* as medication to prevent and control infection outbreaks in pig herds [28]. The spread of resistant strains may be encouraged by prolonged historic prophylactic and metaphylactic use in pig farms [29], which increases selective pressure for antimicrobial-resistant bacteria [30,31]. European data from 2021 to 2022 reported high levels of AMR in NTS from human infections and food production to AMP, TETs and sulfonamides [32,33].

Multidrug resistance (MDR) is defined as resistance to three or more antimicrobial classes [34] and has been widely reported in the livestock industry in Southeast Asia [35,36], inlcuding in Thailand [37, 38, 39]. The dissemination of MDR *Salmonella* through the food chain poses a significant threat to human and animal health, exacerbating morbidity and mortality rates [40]. The global priority list of pathogens was published by the WHO, for which new antibiotics are urgently needed. Several Gram-negative bacteria were included because of their resistance to multiple antibiotic classes, including *Salmonella* spp., which was classified among the high-priority pathogens [41]. Understanding the antibiotic resistance profile of *Salmonella* infections in pigs is crucial for effective surveillance and MDR mitigation. Our study aimed to investigate the prevalence of *Salmonella* spp. among the swine production chain in Southern Thailand, encompassing farms, abattoirs and retail markets. We also characterized their phenotypic AMR profiles, identifying extremely high levels of MDR. Our findings contribute to regional surveillance efforts and provide essential public health data to guide interventions in the pork production chain.

## Methods

### Specimen collection

**Fig. 1.**
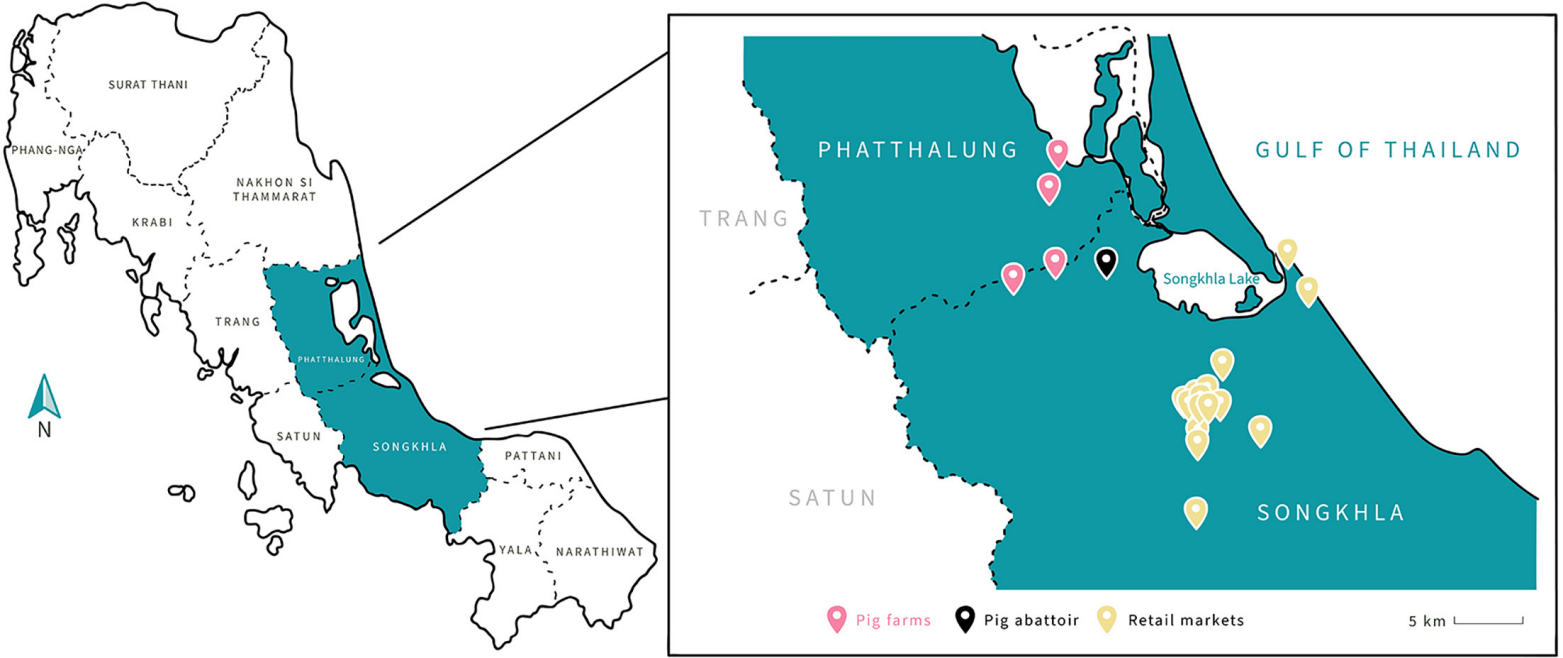
The sampling locations in Phatthalung and Songkhla Provinces, Thailand (pink pin: pig farms, black pin: abattoir, yellow pin: retail markets).

In total, 485 samples were collected from pig farms, an abattoir and retail pork products from two provinces in Southern Thailand. Fresh pig faeces were collected from 150 pigs on four different pig farms (farm A–D), 185 samples from pig abattoir and 150 pieces of pork from retail markets located in Phatthalung and Songkhla provinces (Fig. 1) between 2019 and 2024. Samples were cultured from fresh pig faeces collected from the floor in the stable of each group of pigs, including boars, sows, nursery pigs and fattening pigs. Three different sample types were collected from abattoir: faeces from lairage (*n*=15), swabs from carcasses (*n*=70) and surrounding environment and equipment (*n*=100). Carcass swabs were collected by randomly swabbing a 100 cm^2^ (10×10 cm) area of the carcass; a smaller 25 cm^2^ (5×5 cm) area was swabbed to collect samples from the surrounding environment and equipment (floor, knives, saws, boots, aprons, tables and baskets). All faecal specimens were placed in individual sterile containers; and all swabs were kept in Cary-Blair transport medium (HiMedia, India) and transported in insulated boxes with ice packs to the laboratory within 4 h for *Salmonella* identification.

### *Salmonella* isolation and identification

All presumptive *Salmonella* spp. isolates were cultured and isolated using the ISO 6579-1 : 2017 [42] standard protocol.

#### Faecal samples

Faecal samples from pigs at the four pig farms were collected during the regular daily work routine to minimize any additional stress on the animals. Fresh faecal samples were collected immediately following observation of pig’s bowel movements and stored in individual sterile containers. Once in the laboratory, samples were enriched by placing ~25 g of faeces in 225 ml of sterile buffered peptone water (BPW; Oxoid, England), homogenized in a stomacher for 2 min and then incubated for 18±2 h at 37±1 °C. Selective enrichment for *Salmonella* spp. was achieved by aliquoting 0.1 ml of incubated BPW and three drops spotted on the surface of modified semisolid Rappaport-Vassiliadis (MSRV; Oxoid, England) medium with novobiocin supplement and incubated for 24±3 h at 41.5±1 °C. Selective plating by spreading a 1 µl sterile loop on positive MSRV media is recommended for samples for primary isolation, with cultures that form a grey-white turbid zone extending out from the inoculated droplet confirmed by subculturing onto xylosine lysine deoxycholate (XLD; Oxoid, England) medium and on brilliant green phenol red lactose sucrose (BPLS; Oxoid, England) and incubated for 24±3 h at 37±1 °C.

#### Abattoir samples

Faecal samples from lairage were cultured following the same protocols as the samples from pig farms. However, the non-selective pre-enrichment of swab samples from carcass, environment and equipment were submerged in 10 ml of BPW and incubated for 18±2 h at 37±1 °C; transferred 0.1 and 1 ml of incubated BPW to 10 ml of Rappaport-Vassiliadis soya peptone broth (RVS; Oxoid, England) and 10 ml of Muller–Kauffmann Tetrathionate-Novobiocin Broth (MKTTn; Oxoid, England), respectively; and incubated inoculated RVS broth at 41.5±1 °C for 24±3 h and inoculated MKTTn at 37±1 °C for 24±3 h. One loopful each of RVS and MKTTn was streaked on both XLD and BPLS and incubated at 37±1 °C for 24±3 h.

#### Pork meat samples

Samples from pork meat were subjected to pre-enrichment in BPW, following the same procedure as the faecal samples. Selective enrichment was achieved by transferring 0.1 and 1 ml of incubated BPW to 10 ml of RVS (Oxoid, England) and 10 ml of MKTTn (Oxoid, England), respectively. Cultures inoculated in RVS broth were incubated at 41.5±1 °C for 24±3 h and those inoculated in MKTTn at 37±1 °C for 24±3 h. One loopful each of RVS and MKTTn was streaked onto both XLD and BPLS media and incubated at 37±1 °C for 24±3 h.

All suspected colonies that demonstrated a red colour with a black centre on XLD, and a red centre surrounded by a bright red zone on BPLS, were sub-cultured into nutrient broth (NB; Oxoid, England) and incubated at 37±1 °C for 24±3 h. Presumptive *Salmonella*-positive samples were confirmed by PCR prior to *Salmonella* serotyping.

### DNA extraction

DNA was extracted from all *Salmonella* isolates using the boiling method [43]. Briefly, 1 ml of incubated NB was transferred to a sterile microcentrifuge tube. The cultured sample was centrifuged for 5 min at 14 000 ***g***, and the supernatant was discarded. A second 1 ml of culture was then added to the pellet and centrifuged again at 14 000 ***g*** for 5 min. The supernatant was again discarded, and 600 µl of sterile DNase-RNase-free distilled water was added and centrifuged for 5 min at 14 000 ***g***. After discarding the supernatant, an additional 200 µl of DNase-RNase-free sterile distilled water was added and boiled in a heating block at 100 °C for 10 min and immediately chilled on ice for 5 min. The suspension was centrifuged for 5 min at 14 000 ***g*** at 4 °C, and the supernatant was transferred to a new sterile microcentrifuge tube for storage at −20 °C until required for PCR.

### *Salmonella* confirmation by PCR

Molecular confirmation of *Salmonella* spp. was performed by PCR. The genus-specific genes, *invA* gene (F; GTG AAA TTA TCG CCA CGT TCG GGC AA, R; TCA TCG CAC CGT CAA AGG AAC C) [44] and *iroB* gene (F; TGC GTA TTC TGT TTG TCG GTC C, R; TAC GTT CCC ACC ATT CTT CCC), were used for identification of *S. enterica* [43]. The PCR final reaction volume was 25 µl, consisting of 12.5 µl of OmniPCR Supermix (Bio-Helix, Taiwan), 0.5 µl each of forward and reverse primers (Macrogen, Korea), 6.5 µl of sterile distilled water and 5 µl of DNA sample. The PCR conditions were as follows: initial denaturation at 95 °C for 5 min, followed by 34 amplification cycles of denaturation at 95 °C for 30 s, annealing at 61 °C for *invA* primer set and 55 °C for *iroB* primer set for 30 s, extension at 72 °C for 1 min and final extension at 72 °C for 5 min. Gel electrophoresis was performed using 1.5% w/v of agarose gel (HydraGene, China) and 0.5X Tris-acetate-EDTA (TAE) buffer at 100 V for 25 min. Serotyping of all *Salmonella*-positive samples was identified by the Kauffmann–White scheme at the WHO National *Salmonella* and *Shigella* Center (Nonthaburi, Thailand).

### Antimicrobial susceptibility testing

Susceptibility to ten different antimicrobial agents for all *Salmonella* isolates was determined by disc diffusion methods using Mueller–Hinton agar (Oxoid, England), including AMP (10 µg), amoxycillin/clavulanate (AMC) (20/10 µg), CHL (30 µg), CIP (5 µg), nalidixic acid (NAL) (30 µg), norfloxacin (NOR) (10 µg), streptomycin (STR) (10 µg), sulfisoxazole (SX) (250 µg), TET (30 µg) and trimethoprim/sulfamethoxazole (SXT) (1.25/23.75 µg). Inhibition zones and breakpoints were measured according to the guidelines of the Clinical and Laboratory Standards Institute [45]. *Escherichia coli* ATCC 25922 was used as an internal quality control.

## Results

### Prevalence of *Salmonella*-positive and serotype distribution

*Salmonella* prevalence was observed in 69% (103/150) of samples from pig farms, 46% (85/185) from the abattoir, and 51% (76/150) from retail markets. Among the 103 *Salmonella* isolates from pig farms, 80%(60/75) were from Patthalung and 57% (43/75) from Songkhla. The highest prevalence was on farm B (84%, 21/25), followed by farm C (78%, 39/50), farm D (64%, 16/25) and farm A (54%, 27/50). In the abattoir, *Salmonella* was found in pig faeces (40%, 6/15), pig carcasses (44%, 31/70) and equipment/environmental samples (48%, 48/100). In retail markets, *Salmonella* prevalence was 16% (4/25) in supermarkets and 58% (72/125) in local markets.

Among the 264 *Salmonella* isolates, 18 serotypes were identified (Table 1). The five predominant serotypes were Rissen (39%), Typhimurium (14%), Weltevreden (10%), Stanley (8%) and Lexington (8%). Serotype Rissen showed the highest prevalence among isolates from pig farms (20%), abattoir (51%) and pork in retail markets (53%). Seven serotypes were found across all production chain steps, including the monophasic Typhimurium 4,5,12:i:-, Lexington, Panama, Rissen, Stanley, Typhimurium and Weltevreden. Additionally, six serotypes were exclusively isolated from pig farms: S. 1,4,5,12:i:-, Albany, Derby, Emek, Kedougou, Krefeld and London.

**Table 1.** Serotype distribution of *Salmonella* isolated from swine production chain

	Pig farms	Abattoir	Retail markets	Overall
	No. of isolates (%)	No. of isolates (%)	No. of isolates (%)	No. of isolates (%)
***S.* 1,4,5,12:i:-**	1 (0.97)	0 (0.00)	0 (0.00)	1 (0.38)
***S.* 4,5,12:i:-**	6 (5.83)	1 (1.18)	2 (2.63)	9 (3.41)
***S.* Albany**	1 (0.97)	0 (0.00)	0 (0.00)	1 (0.38)
***S.* Anatum**	3 (2.91)	0 (0.00)	3 (3.95)	6 (2.27)
***S.* Brunei**	7 (6.80)	0 (0.00)	1 (1.32)	8 (3.03)
***S.* Derby**	1 (0.97)	0 (0.00)	0 (0.00)	1 (0.38)
***S.* Emek**	1 (0.97)	0 (0.00)	0 (0.00)	1 (0.38)
***S.* Enteritidis**	11 (10.68)	0 (0.00)	1 (1.32)	12 (4.55)
***S.* Kedougou**	1 (0.97)	0 (0.00)	0 (0.00)	1 (0.38)
***S.* Krefeld**	2 (1.94)	0 (0.00)	0 (0.00)	2 (0.76)
***S.* Lexington**	8 (7.77)	5 (5.88)	7 (9.21)	20 (7.58)
***S.* London**	1 (0.97)	0 (0.00)	0 (0.00)	1 (0.38)
***S.* Newport**	2 (1.94)	0 (0.00)	1 (1.32)	3 (1.14)
***S.* Panama**	5 (4.85)	1 (1.18)	3 (3.95)	9 (3.41)
***S.* Rissen**	21 (20.39)	43 (50.59)	40 (52.63)	104 (39.39)
***S.* Stanley**	10 (9.71)	6 (7.06)	5 (6.58)	21 (7.95)
***S.* Typhimurium**	17 (16.50)	15 (17.65)	6 (7.89)	38 (14.39)
***S.* Weltevreden**	5 (4.85)	14 (16.47)	7 (9.21)	26 (9.85)

### AMR profiles of *Salmonella* isolates

**Fig. 2.**
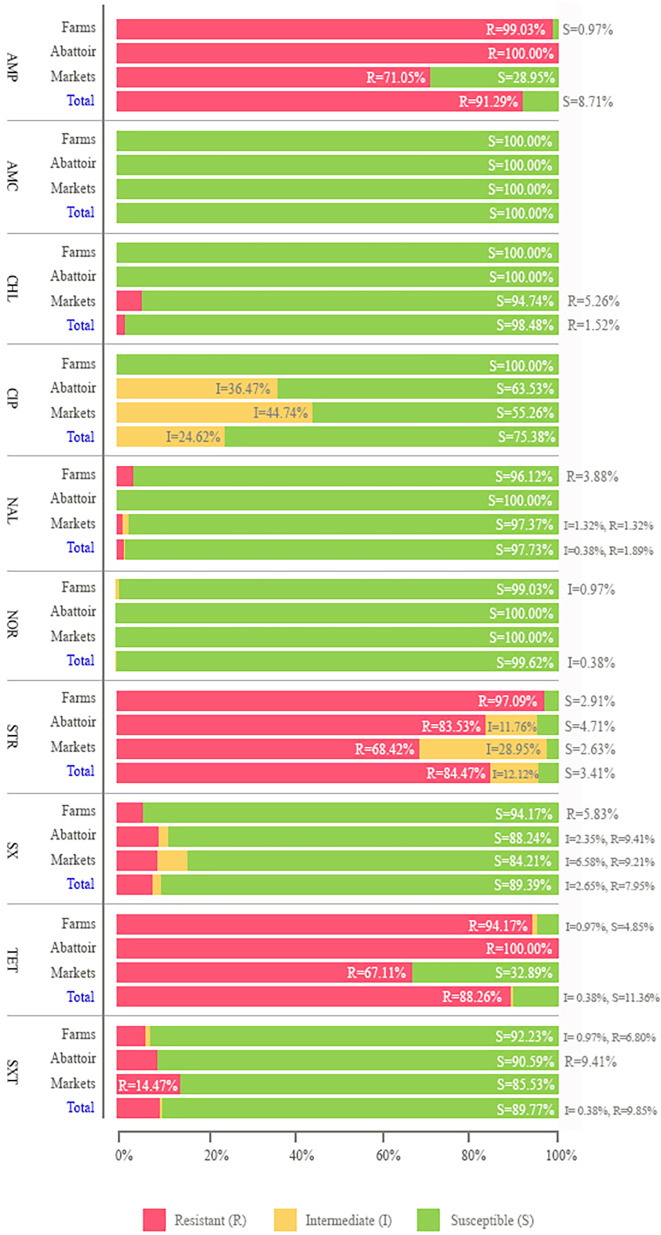
The results of antimicrobial susceptibility testing of *Salmonella* isolates (*n*=264). Each stack column of each bar chart showed the percentage of susceptible, intermediate and resistant of ten antimicrobial agents.

The susceptibility of all the 264 *Salmonella* isolates was tested against ten antibiotics (Fig. 2). Antibiotic agents from seven antimicrobial classes were assessed, including beta-lactam (AMP and AMC), phenicol (CHL), quinolone (CIP, NAL and NOR), aminoglycoside (STR), sulfonamide (SX), TET and trimethoprim (SXT). All isolates were susceptible to AMC, while isolates from pig farms showed susceptibility to AMC, CHL and CIP. Abattoir isolates were susceptible to AMC, CHL, NAL and NOR, and retail market isolates to AMC and NOR. Three isolates from pig farms and retail markets were susceptible to all ten antimicrobial agents (SK_D_PH_SL4, P45 and P61; Table S1). AMP resistance was observed in over 90% of *Salmonella* isolates, most observed in the pig production chain. All abattoir isolates were resistant to AMP and TET. The predominant resistance phenotypes from farms and markets were AMP (99 and 71%), STR (97 and 68%) and TET (94 and 67%).

Among the isolates, approximately 83% showed MDR profiles, with the most common pattern being AMP-STR-TET (69%; Table 2). This pattern was prevalent across all stages of the pig production chain. MDR prevalence was highest in farm isolates (95%), followed by those from abattoirs (85%) and retail markets (63%). We observed 12 different MDR profiles, which varied across the production steps, with 8 patterns identified from farm isolates, 3 from abattoir isolates and 6 from retail market isolates. Resistance to seven antimicrobial agents (AMP–CHL–NAL–STR–SX–TET–SXT) was observed in one isolate from retail market pork (Stanley serotype, P21; supplementary data). Additionally, nearly 11% of isolates were resistant to one or two antimicrobial classes, termed non-MDR, with AMP–TET being the most common pattern (Table 2).

**Table 2.** AMR patterns of *Salmonella* isolates from swine production chain

Antimicrobial-resistant pattern	Pig farms	Abattoir	Retail markets	Overall
	No. of isolates (%)	No. of isolates (%)	No. of isolates (%)	No. of isolates (%)
**All susceptible**	1 (0.97)	0 (0.00)	2 (2.63)	3 (1.14)
Total	1 (0.97)	0 (0.00)	2 (2.63)	3 (1.14)
Non-MDR				
AMP	0 (0.00)	0 (0.00)	2 (2.63)	2 (0.76)
STR	0 (0.00)	0 (0.00)	3 (3.95)	3 (1.14)
AMP-STR	3 (2.91)	0 (0.00)	1 (1.32)	4 (1.52)
AMP-TET	1 (0.97)	16 (18.82)	3 (3.95)	20 (7.58)
Total	4 (3.88)	16 (18.82)	9 (11.84)	29 (10.98)
MDR				
AMP-STR-SX	1 (0.97)	0 (0.00)	0 (0.00)	1(0.38)
AMP-STR-SXT	1 (0.97)	0 (0.00)	0 (0.00)	1 (0.38)
AMP-STR-TET	82 (79.61)	64 (75.29)	36 (47.37)	182 (68.94)
AMP-TET-SXT	1 (0.97)	0 (0.00)	0 (0.00)	1 (0.38)
AMP-NAL-STR-TET	3 (2.91)	0 (0.00)	0 (0.00)	3 (1.14)
AMP-STR-SX-TET	5 (4.85)	0 (0.00)	1 (1.32)	6 (2.27)
AMP-STR-TET-SXT	4 (3.88)	0 (0.00)	5 (6.58)	9 (3.41)
AMP-SX-TET-SXT	0 (0.00)	1 (1.18)	0 (0.00)	1 (0.38)
AMP-NAL-STR-TET-SXT	1 (0.97)	0 (0.00)	0 (0.00)	1 (0.38)
AMP-STR-SX-TET-SXT	0 (0.00)	7 (8.24)	2 (2.63)	9 (3.41)
AMP-CHL-STR-SX-TET-SXT	0 (0.00)	0 (0.00)	3 (3.95)	3 (1.14)
AMP-CHL-NAL-STR-SX-TET-SXT	0 (0.00)	0 (0.00)	1 (1.32)	1 (0.38)
Total	98 (95.15)	72 (84.71)	48 (63.16)	218 (82.58)

## Discussion

Contamination by *Salmonella* spp. was identified at the onset of the production chain, with a notable prevalence of *Salmonella*-positive samples found in pig farms in this study (69%). This prevalence exceeds that reported in the northern region in 2006 (6%) [46], 2014 (35%) [47] and 2020 (22%) [48]. Discrepancies in prevalence could stem from variations in factors such as herd size, farming methods (continuous flow/batch farrowing), sanitation, hygiene practices and regional environmental conditions [49,50].

In the abatoir we studied, we returned a prevalence rate for *Salmonella* of 45% - similar to rates observed in Central Thailand (48%) [51]. The slaughtering process also has a role in spreading *Salmonella* contamination to carcasses, with various factors contributing to increased spread within pig abattoirs, including shedding in pig faeces (40%), which can spread to other pigs within lairages. Additionally, carcass swabs showed a significant *Salmonella* presence (44%), possibly due to gut content spillage during the evisceration step of the slaughter process [52]. Nearly half (48%) of samples from equipment and the abattoir environment were contaminated with *Salmonella*, suggesting that improved hygiene could be a key contributor to reducing the spread of *Salmonella* within the pork production process. Compared with previous studies in the same area (82%, reported in 2013), *Salmonella* contamination of pork produce was at a lower rate (51%) [53], which suggests that there has been an improvement in hygienic practices within local markets over the past decade.

Global concerns regarding the persistence and dissemination of NTS in swine production are underscored by evidence of genetic relationships among *Salmonella* isolates recovered from various points in the production chain [17,54,57]. In this study, *Salmonella* prevalence was highest in pig farms (69%), decreased at abattoirs (46%) and rose again at retail markets (51%), indicating the circulation of *Salmonella* strains within herds and subsequent contamination during processing and retail. Prevention and control strategies targeting infection at the farm level, stringent hygiene protocols at abattoirs and implementation of good sanitation practices in retail markets are crucial for reducing *Salmonella* contamination along the pork production chain [58,61].

*Salmonella* serotypes in human infections vary by geographic region, time and infection source [46,62]. Our findings revealed serotype Rissen as the most frequent serotype, consistent with reports from Europe, Asia, and the USA [51,54, 63, 64]. Serotypes Typhimurium and Weltevreden also ranked highly, in line with reports from South and Southeast Asia [65,67]. While NTS infection in healthy pigs may not cause severe illness, it can affect growth performance, leading to economic losses in the pig production industry. Consequently, antimicrobial agents are often used, with beta-lactams, aminoglycosides and sulfonamides combined with trimethoprim, quinolones and polymyxins being commonly employed in Thailand [27,68]. However, high resistance rates were observed for AMP (91%) and STR (84%), two antibiotics that are commonly used to treat diarrhoea in pigs [68].

Despite TET not being commonly used to treat diarrhoea in pigs, high resistance to TET (88.26 %) was reported in our study, which is consistent with other studies from Thailand (central, northern and northeastern regions), Laos, Cambodia and China [51,62, 66, 67]. This can be explained by the use of TET as a growth promotor in livestock animals since the 1940s worldwide [69], and the use of antibiotics as a growth promoter in livestock animals has been prohibited by the Ministry of Agriculture and Cooperatives in Thailand since 2015. Previous widespread usage of TET in food-producing animals has contributed to strong selection for TET resistance genes, which have become fixed in the population [70]. The high prevalence of AMR in this study reflected that these groups of antibiotics were extensively used in pig production in this area.

The prevalence of MDR *Salmonella* was notably high (83%), with various MDR patterns observed, indicating persistent exposure to antibiotics. High rates of MDR *Salmonella* were present in every step of the production chain (95% from pig farms, 85% from abattoirs and 63% from pork products). Elsewhere in Southeast Asia, there are high levels of MDR, including 98 % in Laos, 70% in Vietnam and 52% in Cambodia [18,71, 72]. Twelve of the MDR patterns observed included co-resistance to AMP–STR–TET, which was the most common MDR profile recovered from each step of the production chain (Table 2), which have all been used frequently in pig farming [73].

## Conclusion

Addressing *Salmonella* contamination in the pig production chain requires a multifaceted approach that includes improved farm management practices, stringent hygiene standards in abattoirs and retail stores and enhanced surveillance for AMR. The high rate of MDR underscores the urgent need for measures to mitigate the spread of MDR *Salmonella* and reduce public exposure to MDR isolates through the pork production chain. Monitoring MDR *Salmonella* in animal-derived foods is crucial for preventing therapeutic failure in both humans and animals, highlighting the importance of ongoing surveillance and intervention efforts in this area.

## supplementary material

10.1099/jmm.0.001894 Table S1.

## Abbreviations

AMC: amoxycillin/clavulanate
AMP: ampicillin
AMR: antimicrobial resistance
BPLS: brilliant green phenol red lactose sucrose
BPW: buffered peptone water
CHL: chloramphenicol
CIP: ciprofloxacin
DNA: deoxyribonucleic Acid
MDR: multi-drug resistance
MKTTn: Muller-Kauffmann Tetrathionate-Novobiocin Broth
MSRV: modified semisolid Rappaport-Vassiliadis
NAL: nalidixic acid
NB: nutrient broth
NOR: norfloxacin
NTS: non-typhoidal *Salmonella*
PCR: polymerase chain reaction
RVS: Rappaport-Vassiliadis soya peptone broth
STR: streptomycin
SX: sulfisoxazole
SXT: trimethoprim/sulfamethoxazole
TAE: tris-acetate-EDTA
TET: tetracycline
WHO: World Health Organization
XLD: xylosine lysine deoxycholate
